# Heart Rate, Stress, and Occupational Noise Exposure among Electronic Waste Recycling Workers

**DOI:** 10.3390/ijerph13010140

**Published:** 2016-01-19

**Authors:** Katrina N. Burns, Kan Sun, Julius N. Fobil, Richard L. Neitzel

**Affiliations:** 1 Department of Environmental Health Sciences, University of Michigan, 1415 Washington Heights 6611 SPH I, Ann Arbor, MI 48109, USA; bkatrina@umich.edu (K.N.B.); kansun@umich.edu (K.S.); 2 Department of Biological, Environmental and Occupational Health Sciences, University of Ghana-Legon, P.O. Box LG 13, Legon, Ghana; jfobil@ug.edu.gh

**Keywords:** electronic waste recycling, occupational health, noise exposure, stress, heart rate

## Abstract

Electronic waste (e-waste) is a growing occupational and environmental health issue around the globe. E-waste recycling is a green industry of emerging importance, especially in low-and middle-income countries where much of this recycling work is performed, and where many people’s livelihoods depend on this work. The occupational health hazards of e-waste recycling have not been adequately explored. We performed a cross-sectional study of noise exposures, heart rate, and perceived stress among e-waste recycling workers at a large e-waste site in Accra, Ghana. We interviewed 57 workers and continuously monitored their individual noise exposures and heart rates for up to 24 h. More than 40% of workers had noise exposures that exceeded recommended occupational (85 dBA) and community (70 dBA) noise exposure limits, and self-reported hearing difficulties were common. Workers also had moderate to high levels of perceived stress as measured via Cohen’s Perceived Stress Scale, and reported a variety of symptoms that could indicate cardiovascular disease. Noise exposures were moderately and significantly correlated with heart rate (Spearman’s ρ 0.46, *p* < 0.001). A mixed effects linear regression model indicated that a 1 dB increase in noise exposure was associated with a 0.17 increase in heart rate (*p*-value = 0.01) even after controlling for work activities, age, smoking, perceived stress, and unfavorable physical working conditions. These findings suggest that occupational and non-occupational noise exposure is associated with elevations in average heart rate, which may in turn predict potential cardiovascular damage.

## 1. Introduction

Since the 1980s continuing and rapid technological advances have made the functional life of technologies relatively short. By 2002, the rate of PC obsolescence exceeded the rate of production, creating a need for recycling of the obsolete equipment, variously known as electronic waste or e-waste [1]. E-waste has been defined as discarded nonworking electronic products such as cell phones, computers, televisions, and appliances, or other such working products that are no longer considered useful by the owner or manufacturer [2]. In 2014 alone, approximately 41.8 million metric tons of e-waste were generated worldwide [2], and the generation of e-waste is expected to continue to increase over time. E-waste recycling has emerged as an important green industry, the primary goals of which are to remove components from electronic devices for reuse, and to prevent toxic heavy metals from entering sanitary landfills. Economic pressures in high-income countries, including higher wages and better worker protection, have resulted in the movement of e-waste recycling activities to low- and middle-income countries [3], though waste generation and treatment without movement to other countries is also increasing [4]. 

Wastes exported to lower-income countries are dismantled for recycling using crude manual tools and techniques [5], which creates important economic opportunities in the recipient countries while simultaneously creating hazards to human health and the environment. Only a handful of studies have assessed occupational health risks associated with e-waste recycling. Heavy metal exposures, including mercury, cadmium, and lead, as well as exposures to other chemical contaminants, have been the focus of most of the existing literature on this topic [6,7,8]. However, e-waste workers also have potential exposures to high levels of physical agents such as noise while they dismantle and shred electronic components [9]. While noise is a well-understood cause of hearing loss [10], several studies have shown evidence that noise exposure is associated with increased heart rates [11,12], which can in turn result in chronic and acute conditions such as hypertension and myocardial infarction [10]. The mechanism underlying this relationship is unknown, but may involve a general stress reaction during occupational noise and/or disruption of sleep due to noise [10]. There is additional evidence that variability in noise levels in particular exerts a negative effect on heart rate variability, which is also indicative of increased risk of cardiovascular disease [13,14]. Additionally, exposures to mercury, cadmium, and lead may be an independent risk factor for cardiovascular disease [15]. While there is some evidence of an association between each of these exposures and cardiovascular health effects, most of these have been evaluated in epidemiological studies of chronic (e.g., months or years-long) exposures; evidence is lacking regarding short-term (e.g., hours to days) exposures and among transient workforces.

Working conditions at the informal e-waste recycling sites in low- and middle-income countries are communal and often include both work- and non-work-related activities, such as socializing with friends and family, meals and breaks, and prayer. The occurrence of these activities at potentially contaminated sites may be particularly important for the young adults and children who live adjacent to, or in some cases on, the recycling sites [16,17], as was the case for the large Agbogbloshie e-waste recycling site in Accra, Ghana [18].

Our study had three goals. The first was to characterize the noise levels experienced by e-waste workers at the Agbogbloshie e-waste recycling site. The second was to examine the association between occupational noise exposures and heart rates among these workers over a short period (one day). The final goal was to evaluate the potential influence of work activities and perceived stress on the observed relationship between occupational noise exposures and heart rates over a short period. Most previous studies have failed to adequately control for work activity in assessments of the relationship between noise exposure and heart rate or other surrogates for cardiovascular impact of noise [19,20,21], even though physical workload has a strong potential to confound this relationship. 

## 2. Experimental Section

### 2.1. Overview

The focus of this analysis was a dataset collected as part of a cross sectional study conducted in May 2014 on e-waste workers at Agbogbloshie. Other data collected at this time are described elsewhere [18]. All subjects gave their informed consent for inclusion before they participated in the study. The study was conducted in accordance with the Declaration of Helsinki, and the protocol was approved by the University of Michigan Institutional Review Board (HUM00084062) and the University of Ghana Institutional Review Board at the Noguchi Memorial Institute for Medical Research (NMIMR-IRB CPN 070/13-14). Data were collected by a research team composed of students, staff, and faculty members from the University of Michigan and the University of Ghana-Legon.

### 2.2. Site Description and Recruitment 

**Figure 1:**
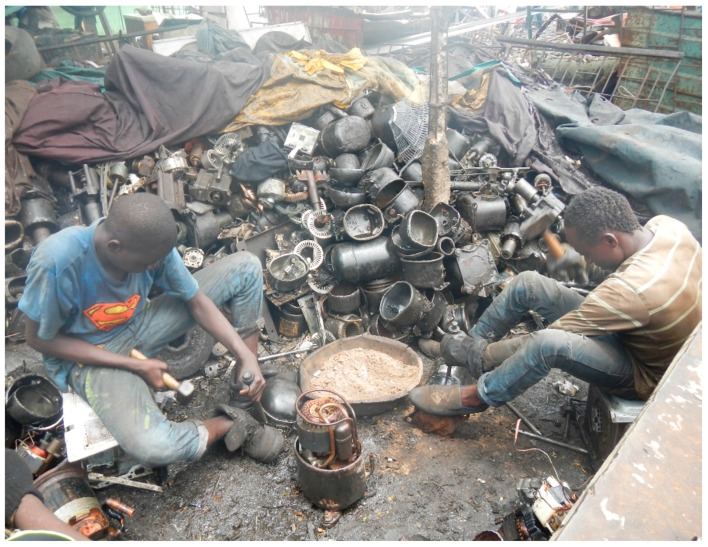
E-waste dismantling activities at Agbogbloshie.

Workers at the site were involved in a variety of e-waste recycling activities. These included: collecting e-waste; sorting and distributing bulk incoming e-waste; dismantling reusable electronic components; manually breaking down non-reusable e-waste (typically with hammer and chisel, as shown in Figure 1); sorting broken down e-waste materials; and stripping coatings and plastics from desirable e-waste metals such as copper (typically using a burning rubber tire as a heat source to melt the coatings). The work was conducted outdoors, often without overhead cover or other protection from the elements and in close proximity to other workers conducting related activities. The worksite had no improved drinking water supply and crude and inadequate sanitation facilities. Rudimentary worker housing constructed of salvaged materials and rubbish was interspersed with some work areas. Off-duty workers, friends, and family were often observed socializing with workers actively conducting e-waste recycling activities at the site. 

Male e-waste workers aged 18 years or greater and onsite at the time of recruitment (though not necessarily actively working) were eligible to participate in the study. Recruitment was conducted with the approval and assistance of the Chairman of the Greater Accra Scrap Dealers Association, who nominally oversaw all e-waste and metal scrap recycling operations at Agbogbloshie. Three Ghanaian translators assisted with recruitment and data collection efforts. Male e-waste workers aged 18 and over participated in this study over a single day each, and, as with recruitment, were not required to be actively working on their day of participation. Each participant was approached in person during their normal work activities and read a recruitment script; interested individuals were then given an informed consent form. Both the script and the consent form were read to potential subjects in their native language. Interested volunteers signed or provided an ink thumbprint on the informed consent form and were enrolled in the study. Participants were given 9 GHS (3 USD) and a 3 GHS (1 USD) snack as a thank-you for their participation, and received no-cost transportation to and from the nearby Ghana Post Clinic where a medical exam and other aspects of the study not described here were conducted. 

### 2.3. Questionnaire and Activity Log

A questionnaire was administered to subjects by the research team via the three trained interpreters, and the surveys were filled out for the participants by the research team. Questionnaire items addressed demographic information (e.g., age, education, marriage, income, and living arrangements); exposure to tobacco smoke and alcohol consumption; self-reported health status (e.g., health history, shortness of breath, abnormal heart beat, dizziness, hypertension, medications, hearing ability); perceived noise exposure and annoyance during work and non-work activities; working conditions, including unfavorable physical working conditions; occupational history; and Cohen’s Perceived Stress Scale (PSS), a validated scale of self-reported stress [22]. 

After completing the questionnaire, subjects were provided with an activity log and asked to report the amount of time spent in each of their daily activities (e.g., specific work tasks, sleep, prayers, rest, socializing, *etc.*) with approximately 15-min time resolution over the subsequent 24-h period. The activity log used here was modeled after others used in previous studies of occupational noise exposure in dynamic work environments such as construction [23] and forestry [24], as well as for non-occupational activities [25,26].

### 2.4. Noise Exposure and Heart Rate Measurements

Personal noise exposures were measured for each subject using an ER-200D Personal Noise Dosimeter (Etymotic Research Inc. Elk Grove Village, IL, USA), a small device which approximates the performance of a Type 2 noise dosimeter [27]. The devices were configured to measure the equivalent continuous average noise level (L_EQ_) according to the exposure limit for community noise recommended by the World Health Organization: A-weighting, slow time response, 3 dB time-intensity exchange rate, 70 dB threshold, and 75 dB criterion level [28]. The range of the dosimeter was 70 to 130 dBA. Average and maximum noise exposures were datalogged every 3 min 45 s (the default and only datalogging interval length available for this unit) over up to 24 h of measurement. Heart rates were monitored for up to 24 h on each subject using a RS400 Heart Rate Monitor consisting of a watch and matched wireless chestband combination (Polar Electro, Lake Success, NY, USA). The average heart rate (in beats per min, bpm) was datalogged at 5-s intervals over the same period that the noise dosimeter was worn. Both the dosimeters and heart rate monitors were fit to each subject onsite, and were returned by subjects approximately 24 h later onsite at a prearranged location and time. 

### 2.5. Data Cleaning and Statistical Analysis

SPSS (IBM SPSS Statistics for Windows, Version 23.0, IBM Corp., Armonk, NY, USA), was used to conduct all statistical analyses. Statistical results were considered significant where *p* < 0.05. The datalogged values from the noise dosimeters and heart rate monitors were time-synchronized, and then the 5-s intervals from the heart rate monitors were collapsed to a 3 min 45 s average to match the logging interval of the dosimeters. 

Noise levels, particularly during nighttime periods, were often below the dosimeter threshold of 70 dBA, and were assigned a value of 0 dBA by the dosimeter as a result. These values that were below the limit of detection were certainly above 0 dBA (the complete absence of any audible sound, an impossible level in dense urban environments), and all 0 dBA values were therefore replaced with a value of 49.5 dBA (*i.e.*, 70 dBA threshold divided by $\sqrt{2}$), a standard procedure for replacing values below the limit of detection in environmental sampling [29]). In addition to this limit of detection issue with the noise dosimeters, the heart rate monitors routinely lost their wireless connection to their matched chestband when pulses dropped below 60 bpm (during sleep, primarily). 

Univariate descriptive statistics were computed for all variables, and bivariate relationships were analyzed using visual examination and Spearman’s correlation coefficients. Synchronized noise levels and heart rates were analyzed after stratification by reported work and non-work activity within and across subjects. We also computed the percentage of subject measurements that exceeded relevant exposure guidelines for occupational and total noise exposures—an 85 dBA 8-h time-weighted average [30] and a 24-h average [28], respectively. Variables that did not demonstrate significant bivariate relationships with heart rate or noise were not considered for further analysis, with the exception of *a priori* assumed potential confounders, as described below. 

For each subject we computed a recommended maximum heart rate (220 bpm—subject age) [31], and then assessed whether or not their highest measured average heart rate exceeded this value. We also used Spearman’s correlation coefficients to assess the relationship between highest measured average heart rate and the total number of four cardiovascular-related questionnaire items (frequency of shortness of breath in past two weeks, frequency of dizziness in past two weeks, frequency of abnormal heart beat in past two weeks, diagnosis of hypertension) to which subjects gave a positive (*i.e.*, occurrence of symptom in past two weeks or diagnosis of hypertension) response.

To evaluate the relationships between 3 min 45 s average L_EQ_ noise level—as well as a variety of other predictors and potential confounders—and the matched 3 min 45 s average measured heart rate as the outcome variable, a series of three multivariate linear regression models were created. In each of these models, all matched heart rate and noise exposure data were included as outcome and predictor variables, respectively, along with a random effect for subject ID to account for the repeated measures within individual. Alcohol consumption could not be addressed in these models due to an insufficient number of subjects reporting this activity. In the first multivariate mixed effects model, the potential confounders of age, income, and proximity to smokers were modeled in addition to L_EQ_ noise levels, and noise exposure. In the second model, we included all of the variables from model 1, in addition to information about work-related activities, in order to control for possible confounding from variations in average heart rate due to physical workload associated with each subject’s work. The third model adjusted for all of the variables in model 2, and added perceived stress scores, perceived exposures to loud noise at work and away from work, and perceived exposure to unfavorable physical work conditions, in order to evaluate the degree to which perceptions of the working and non-working environment influenced average heart rate. 

For each of our models, we assessed multiple possible covariance structures, including robust independent, autoregressive first order (AR1, used to describe relationships in an ordered sequence of values), and exchangeable. The final covariance structure was selected using the QIC model selection statistic (the information criterion for quasi-likelihood under the independence model), with lower QIC values indicating better model fit [32]. 

## 3. Results

### 3.1. Demographics

We recruited 57 e-waste workers. The average age of all the workers was approximately 25 ± 8 years old; subjects ranged in age from 18–61 (Table 1). Most subjects were married, had no or little education, and had a daily income <3 GHS (1 USD). Subjects had worked an average of 10–11 h per day for an average of 6 years at the Agbogbloshie site. Nine subjects (15.8%) reported currently smoking cigarettes, and smoked an average of 11 ± 11 cigarettes per day, with an average smoking history of 11 ± 7 years. Nearly all subjects reported being around smokers at work. Only one subject reported alcohol consumption. The majority of subjects indicated moderate to high levels of stress, and were reported exposure to loud or annoying noises at work and at home. The PSS results suggested moderate to high levels of stress (mean PSS score 25 out of 40 possible points, with higher stress scores indicating greater stress). Diagnoses of hearing loss and heart disease were rare among sampled subjects. Fifteen workers (26.3%) reported difficulty hearing. A number of subjects reported experiencing abnormal heartbeats and other potential indicators of CVD (e.g., shortness of breath, exhaustion, and dizziness); 11 (19.3%) responded positively to all four cardiovascular-related items. Two subjects (3.5%) exceeded their maximum target heart rate during monitoring. The correlation between the maximum measured heart rate and number of cardiovascular-related questions answered positively (e.g., shortness of breath in past two weeks, dizziness in past two weeks, abnormal heart beat in past two weeks, diagnosis of hypertension) was significant (Spearman’s ρ 0.31, *p* = 0.019), suggesting that subjects with the highest heart rates also reported the most cardiovascular symptoms. 

**Table 1:** Demographics and self reported health status (*N* = 57 subjects).

Variable	N	Mean	SD
Age (years)	57	25.8	7.9
Daily work duration (hours)	53	10.5	2.2
Work experience (years)	53	5.8	3.9
Daily income (GHS)	57	2.3	1.4
Duration lived at Agbogbloshie (years)	57	6	4.2
PSS score	53	25	5.2
	**Category**	**N**	**%**
Sleep near Agbogbloshie		56	98.2
Marital status	Single	22	38.6
	Married	32	56.1
	Divorced/Separated	3	5.3
Education	None	31	54.4
	Primary	11	19.3
	Middle/JSS	12	21.1
	Secondary/SSS	3	5.3
Experience tinnitus	Never	13	22.8
	Almost never	5	8.8
	Sometimes	21	36.8
	Fairly often	4	7.0
	Very often	14	24.6
Experience exhaustion after work	Sometimes	4	7.0
	Almost always	21	36.8
	Always	31	54.4
Experienced shortness of breath or difficulty in breathing in last two weeks	Never	21	36.8
	Almost never	6	10.5
	Sometimes	21	36.8
	Almost always	5	8.8
	Always	4	7.0
Experienced dizziness over the last two weeks?	Never	15	26.3
	Almost never	6	10.5
	Sometimes	25	43.9
	Almost always	7	12.3
	Always	4	7.0
Experienced heart beating abnormally over the last two weeks?	Never	10	17.5
	Almost never	4	7.0
	Sometimes	26	45.6
	Almost always	9	15.8
	Always	8	14.0
Diagnosed with high blood pressure		7	12.3
On medication		1	1.8
Experience difficulties hearing		15	26.3
Diagnosed with hearing loss?		2	3.5

### 3.2. Self-Reported Exposures

Table 2 shows self-reported exposures to noise, unfavorable working conditions, and insufficient income. Fifty-five subjects (96.5%) reported working in high noise, with an average duration of 6 ± 4 years. None of these subjects reported ever wearing hearing protection during noise at or away from work. The majority of subjects (77.2%) reported a little or a great deal of annoyance from noise at work. A similar fraction reported annoyance from noise at night, and 36 (63.1%) reported that their sleep was at least sometimes affected by noise at night. Thirty-five (61.4%) reported always or almost always working in unfavorable physical conditions, and 15 (26.3%) reported always or almost always experiencing violence or harassment at work. Insufficient income to support themselves and their families was also commonly reported. 

**Table 2:** Self-reported exposures (*N* = 57 subjects).

Variable	Category	N	%
Exposed to noise at work		55	95.9
Bothered/annoyed by loud noise at work	Not at all	13	22.8
	A little	24	42.1
	A great deal	20	35.1
Exposed to noise away from work		45	78.9
Bothered/annoyed by noise at night	Not at all	16	28.1
	A little	21	36.8
	A great deal	20	35.1
Sleep affected by noise at night	Never	14	24.6
	Almost never	7	12.3
	Sometimes	19	33.3
	Fairly often	7	12.3
	Very often	10	17.5
Exposed to unfavorable physical conditions at work	Never	2	3.5
	Sometimes	20	35.1
	Almost always	9	15.8
	Always	26	45.6
Experience violence or harassment at work	Never	1	1.8
	Almost never	6	10.5
	Sometimes	34	59.6
	Almost always	10	17.5
	Always	5	8.8
Income insufficient to support self and family	Never	3	5.3
	Almost never	4	7.0
	Sometimes	12	21.1
	Almost always	11	19.3
	Always	25	43.9

### 3.3. Noise Exposure and Heart Rate Measurements

When we evaluated workshift exposures among the 20 subjects who worked at Agbogbloshie during their monitoring period, 10 (43.5%) had equivalent average exposures above the recommended occupational exposure limit of 85 dBA (data not shown). Similarly, among all participating workers, the 24-h equivalent average exposure exceeded the recommended community exposure limit of 70 dBA for 45% of subjects.

**Figure 2:**
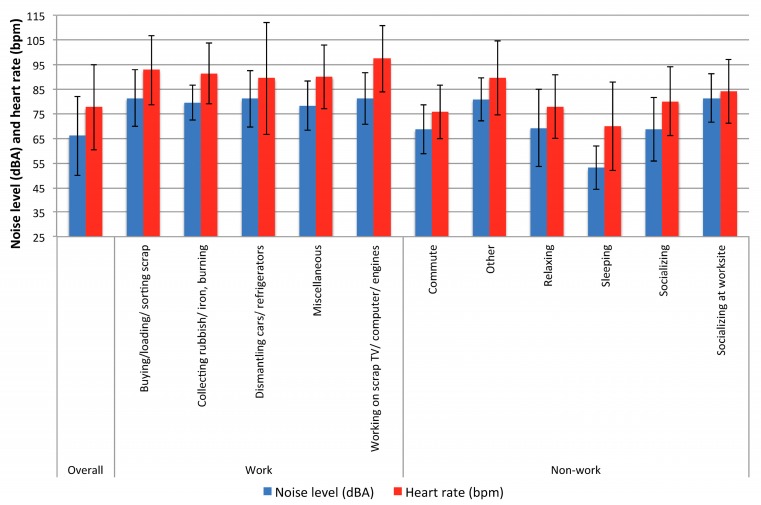
Average noise levels (dBA) and heart rate (bpm) overall and by work and non-work activity (*N* = 57 subjects).

Table 3 shows information for the work activities conducted by the subjects, along with the noise exposures and heart rates measured during these activities. Activity reports were available for all 57 subjects, but not all subjects participated in all activities; hence the number of subjects for all of the individual activities is less than 57. Subjects were monitored for a total of 77,321 min (1288.7 h). One worker who reported working continuously for 24 h was eliminated from this analysis. Dropped connections between the heart rate monitors and their matched chestbands during periods of heart rate below 60 bpm (during evening and nighttime sleeping hours), resulted in the loss of slightly more 5003 min 45 s intervals (*i.e.*, approximately 312 h, or 31% of possible heart rate data ). These periods of lost heart rate data likely represent windows of time with low physical activity and low stress. Work activities were reported by only 20 monitored subjects (35%), three of whom performed multiple work activities. Nonoccupational activities were reported far more commonly, as might be expected for a transient workforce with a variable workload. The majority of subjects reported time spent socializing at Agbogbloshie. The greatest amount of monitoring data were collected on the activities of sleeping, relaxing, and socializing. The highest 3 min 45 s average L_EQ_ noise levels were associated with work activities (dismantling cars or refrigerators; buying, loading, or sorting scrap; and working on scrap TV/computer/car engines) and socializing, while the lowest noise levels were associated with sleeping, relaxing and socializing. Heart rates were highest during work activities (buying, loading, or sorting scrap; collecting rubbish or iron and burning; and working on scrap TV/computer/car engines), and were lowest during sleeping and commuting. Figure 2 shows the association between average 3 min 45 s heart rate and matched average noise level overall and by select work and non-work activities. Spearman correlation coefficients between noise and heart rate were weak to moderate, but highly significant: ρ 0.46 (*p* < 0.001) overall, ρ 0.16 (*p* < 0.001) during work activities, and ρ 0.44 (*p* < 0.001) during non-work activities. 

**Table 3:** Noise exposures and matched heart rates related to work and non-work activities (*N* = 56 subjects).

Activity	N Subjects Reporting	Duratio (min)	Subject Duration (min)		Subject Noise Level (dBA) **		Heart Rate (bpm)	
			Mean	SD	Mean	SD	Mean	SD
Overall	57 *	45098	791.2	604.6	66.0	16.0	77.7	17.3
Occupational	20	4830	966	798.1	80.8	11.0	91.8	17.4
Buying, loading, or sorting scrap	5	1815	363	10.6	81.3	11.4	92.7	14.0
Collecting rubbish or iron, burning	3	274	91.3	29.2	79.6	7.2	91.4	12.5
Dismantling cars or refrigerators	10	1579	157.9	106.1	81.0	11.4	89.4	22.8
Miscellaneous	3	476	158	5.3	78.2	9.9	90.1	13.0
Working on scrap TV/computer/car engines	2	619.0	309.4	204.2	81.1	10.5	97.3	134.
Non-occupational	53	30473	575	509	63.1	15.9	74.9	16.7
Commute	48	2974	48.0	40.0	68.7	10.0	75.7	10.9
Other	3	229	76.4	105.9	80.9	8.8	89.5	15.0
Relaxing	38	7549	199	66.0	69.2	15.6	78.0	13.0
Sleeping	41	15814	386	289	53.0	8.8	70.0	18.0
Socializing	10	1110	111	88.0	68.7	12.9	80.0	14.0
Socializing at the work site	32	5505	172	27.0	81.3	9.8	84.0	13.0

* 56 subjects for work activities, 57 subjects overall. ** Intervals of noise with LEQ levels below the limit of detection were assigned values of 49.5 dBA (*i.e.*, 70/$\sqrt{2}$).

The results of our multivariate mixed effects linear regression models on the 3 min 45 s interval data are shown in Table 4. For these models, we selected the robust independent covariance structure, as it displayed the lowest QIC values of any of the covariance structures evaluated. In all models, three subjects were dropped: one who did not report smoking status, one who reported 24 h of working time, and the single subject who reported consuming alcohol. 

In model 1, which adjusted for L_EQ_ noise exposure and three other potential confounders, only noise exposure was found to be a significant predictor of heart rate; heart rate increased 0.5 bpm (*p*-value = 0.001) for each 1 dB increase in L_EQ_ noise level. 

Model 2, which further controlled for work activity, indicated that working on scrap TV/computer/car engines was the only work activity significantly associated with an increase in heart rate (coefficient = 17.0 bpm, *p*-value < 0.001). However, even after controlling for work activities, L_EQ_ noise level was still significantly associated with increased heart rate (coefficient = 0.3, *p*-value = 0.01). Also in model 2, not working around smokers was associated with a significantly decreased heart rate compared to working around smokers. 

In model 3, which further controlled for perceived exposures to noise, stress, and other factors, the effect of working around smokers was significant compared to not working around smokers, but was reversed from model 2 (coefficient = 11.5 bpm, *p*-value=0.01). No work activities reached statistical significance in model 3, but L_EQ_ noise level remained significant, with a 1 dB increase in noise associated with a 0.2 bpm increase in heart rate (*p*-value = 0.01). A number of perceived exposure factors were significantly associated with changes in heart rate. Subjects who did not report exposure to noise away from work showed a significant decrease in heart rate (coefficient = −30.5 bpm, *p*-value=0.001) compared to those who did. Also, subjects who reported sometimes or almost always working in unfavorable physical conditions had significantly lower heart rates (coefficients of about −20 bpm) compared to workers who always worked in unfavorable physical conditions. PSS score was not significantly associated with changes in average heart rate. 

**Table 4:** Regression models with heart rate as dependent variable (*N* = 54 subjects *).

Parameter	Model 1			Model 2			Model 3		
	Coefficient	SE	*p*-Value	Coefficient	SE	*p*-Value	Coefficient	SE	*p*-Value
Intercept	52.5	5.1	0.001	52.2	17.2	0.00	88.1	34.4	0.01
Around smokers									
No	2.6	1.4	0.06	−20.1	2.2	0.00	11.5	4.7	0.01
Yes	Ref	Ref	Ref	Ref	Ref	Ref	Ref	Ref	Ref
Age (years)	−0.1	0.1	0.3	0.6	0.6	0.3	0.8	0.6	0.15
Income (Cedis)	−1.4	0.8	0.07	−0.3	2.3	.0.9	-2.8	1.9	0.13
L_EQ_ noise level (dBA) **	0.5	0.05	0.001	0.3	0.1	0.01	0.2	0.08	0.01
Work activity									
Dismantling cars or refrigerators				−7.8	7.7	0.3	−11.4	6.5	0.08
Collecting rubbish or iron, burning				−0.3	6.1	1.0	4.1	6.2	0.51
Buying, loading, or sorting scrap				−0.6	7.6	0.9	−12.1	7.0	0.08
Working scrap TV/computer/car engines				17.0	5.4	0.00	−4.1	7.2	0.60
Miscellaneous				Ref	Ref	Ref	Ref	Ref	Ref
Noise away from work									
No							−30.5	6.7	0.001
Yes							Ref	Ref	Ref
Exposed to unfavorable physical work conditions									
Sometimes							−20.2	3.4	0.001
Almost always							−20.9	7.0	0.003
Always							Ref	Ref	Ref
PSS score							−0.3	0.74	0.7

* One subject reporting 24 h of work time was dropped from analysis, along with one subject who did not report smoking status and the single subject who reported consuming alcohol. ** Intervals of noise with L_EQ_ levels below the limit of detection were assigned values of 49.5 dBA (*i.e.*, 70/$\sqrt{2}$).

## 4. Discussion

We were able to achieve all three goals of our study, namely characterization of the noise levels experienced by e-waste recycling workers at Agbogbloshie, examination of the association between occupational noise exposures and heart rates among these workers, and evaluation of the potential influence of work activities and perceived stress on the observed relationship between occupational noise exposures and heart rates. Our results indicate that workers had the potential for exposure to noise that frequently exceeds recommended occupational and community noise exposures, and that self-reported hearing difficulties were relatively common among participating workers. We also demonstrated moderate to high levels of perceived stress among the workers sampled, as well as a number of symptoms (e.g., dizziness, shortness of breath) that may indicate cardiovascular issues. Higher L_EQ_ noise levels were found to be significantly associated with increased heart rate. This relationship remained even after controlling for work activities, age, smoking, alcohol consumption, perceived stress, and unfavorable physical working conditions. Worker who did not report noise exposures outside of work also showed significant reductions in heart rate compared to those who did report such exposures. These findings suggest that noise exposure can produce short-term elevations in average heart rate, which may in turn be a predictor of potential damage to the cardiovascular system following chronic exposures to high noise [33,34].

Only a handful of studies have focused on occupational health and safety among e-waste recycling workers [7,35,36]. Most previous studies have instead focused on the assessment of environmental contamination on and around recycling sites [37,38,39,40,41]. While exposure to hazardous chemicals in the environment is important from an occupational and community health perspective, acute injury risks and physical hazards are equally important. Chronic noise exposures, for example, have been associated with increased risks of noise-induced hearing loss [42], occupational injury [43], fatigue [44], and reduced performance [45], and these health effects may have a more immediate impact on workers than chemical hazards. Our study suggests that physical hazards such as noise are present at sufficient magnitude to warrant further research, and our study therefore represents an important step toward protecting workers in the important and expanding e-waste recycling industry. 

Our results support those of a recent study on Ghanaian artisanal and small-scale gold miners—another occupational group with physically demanding work situations and substantial exposures to noise and heavy metals [46]. Green *et al*. [47] used a mixed effect linear regression model similar to the approach used in the current study and found that a 1 dB increase in L_EQ_ was associated with a statistically significant increase of 0.29 bpm in heart rate, after adjusting for age and sex. The effect size noted by Green et al is consistent with the effects observed here in models 1 through 3, and provides further support to the notion that short-term average heart rate is influenced by noise. Models 2 and 3 in the current study represent an important step forward from the models presented by Green *et al*. [47], since our models adjust for work activity, which is an important confounder in the relationship between noise exposure and heart rate.

We did not collect a resting heart rate, and so have no data to compare directly to resting heart rate guidelines. However, the overall average heart rate measured among our subjects (mean 77.7 ± 17.3 bpm), which includes periods of rest and relaxation, is within the normal adult range of 60–100 bpm [31]. However, two of our subjects exceeded their recommended maximum target heart rate. Nearly 20% of subjects responded positively to all four cardiovascular-related questionnaire items, and we observed a significant correlated between maximum measured heart rate and number of positive cardiovascular questionnaire items, 

We did not find significant relationships between demographic factors (e.g., age, income, *etc.*) and heart rate. It may be that unaccounted-for psychological resiliency factors may attenuate the effects of demographics on heart rates [48]. Likewise, while unfavorable conditions at work such as stress, violence and perceived annoyance can affect heart rate [49], these factors were not uniformly significant in our analyses. Unfavorable physical working conditions that occurred less than always were associated with a substantial reduction in average heart rate when compared to always working under such conditions. However, violence, perceived annoyance, and PSS score were not significantly associated with heart rate. A lack of association between perceived annoyance and heart rate has also been noted in other studies [11]. These findings may again reflect psychological resilience to stress. Also, physical working conditions may directly influence heart rate through modifications in behavior and movement to adapt to the site, whereas annoyance, experience with violence, and perceived stress may have more subtle indirect effects on heart rate. The loss of nighttime and evening heart rate data (*i.e.*, data from periods where both heart rate and perceived stress were likely to be low) limits our ability to quantitatively evaluate the relationship between heart rate and perceived stress during such periods.

Finally, while there is evidence that smoking increases heart rate [50,51] the change in direction of the significant coefficients between our models 2 (negative coefficient) and 3 (positive coefficient) did not provide clear evidence of this relationship among the sampled e-waste recycling workers. Not being around smokers was associated with a large reduction in heart rate in model 2, which adjusted for demographic factors and work activity, while not being around smokers in model 3, which adjusted for those factors plus other perceived stress and exposure factors, was associated with a smaller but positive coefficient. This suggests that factors such as work and perceived stress may play a role in determining heart rate that is independent of smoking, and warrants further evaluation [52,53,54].

### Limitations

There are a number of limitations of this study that may reduce the generalizability of our findings. The first and primary limitation is the sample size of 57 male subjects. Accurate estimates of the size of the workforce at Agbogbloshie are not available, but it was evident during our research that several hundred workers were employed at the site. Our sample size of 57 likely represents a substantial portion of those workers, and so our results may be representative of workers at Agbogbloshie, reputedly one of the largest e-waste recycling sites in the world. However, given that electronic waste recycling sites exist around the globe, in many different cultural, geographic, and social settings [55], the degree to which the noise and stress exposures and their association with heart rate are generalizable to other locations is unclear. The small sample size in this study also influences the effect estimates for the fixed-effect terms in our models, potentially resulting in unstable covariance structures or large variations in effect estimates.

Our dependence on a number of translators due to the range of languages spoken onsite is a second limitation; while these translators were trained on the goals of the study and advised on how to administer the questionnaires, it is possible that the questions—and their responses—were translated incorrectly. This would not alter the relationship we noted between noise and heart rate, but could have introduced information bias into the questionnaire results. Along the same lines, the lack of a positive linear relationship between PSS values and heart rate may have resulted from interpretation of the meaning of the PSS questions by subjects that differed from our own interpretation. 

The third limitation regards the instrumentation used to measure noise exposures and heart rate. True Type 2 [27] noise dosimeters and heart rate monitors capable of measuring inter-beat intervals (IBIs, the time periods between heartbeats) are considered the gold standards for measuring noise exposures [56] and heart rate variability [57], respectively, under field conditions. However, for this pilot study under harsh environmental conditions, more rugged and less expensive units that approximated Type 2 noise measurement performance and measured average HR but not IBI were used to minimize the likelihood of damaged equipment and potential loss of equipment by subjects. While we deemed this an acceptable tradeoff, the instruments used did result in a substantial amount of missing data and data below the limit of detection, which undoubtedly introduced error into our analyses. 

The final limitation relates to potentially unmeasured confounders and covariates that might influence the relationship between noise exposure and heart rate. Heart rate is influenced by many factors, including exposures to certain chemical hazards (e.g., air pollution [58,59] and lead), existing medical conditions (e.g., diabetes, and hyperlipidemia), smoking [60], and alcohol use [61]. While we were able to control for exposure to cigarette smoke, our subjects were certainly exposed to air pollution in their work [36], and some subjects may have had unrecognized medical issues and alcohol use. Particularly in our data, only one individual stated drinking alcohol, and, unsurprisingly, inclusion of alcohol consumption into the model resulted in an insignificant relationship with heart rate when work and stress were added to the model.

## 5. Conclusions

This appears to be the first study to examine occupational noise and its health effects among electronic waste recycling workers. We found that the workers assessed were exposed to potentially harmful levels of both occupational and non-occupational noise. We also found evidence that heart rate was significantly associated with noise. This relationship was observed even after controlling for a number of covariates and potential confounders, including work activity, and did not appear to be modified by perceived stress, as measured using Cohen’s Perceived Stress Scale [22]. The addition of work categories significantly altered the relationship between heart rate and noise, although this relationship remained significant and positive after controlling for work activity. The results of our study suggest that, in addition to being exposed to a wide variety of chemical hazards [36], e-waste recycling workers also have the potential for exposure to physical hazards and associated adverse health outcomes. When examining the noise exposure it is clear that for these workers, the highest noise levels were associated with the work tasks related directly to e-waste processing (Table 3) More research is needed to confirm the relationship between noise exposure and heart rate among these workers, but sufficient evidence already exists to warrant interventions designed to improve working conditions at e-waste recycling sites in low- and middle-income countries.
